# Trehalose induces SQSTM1/p62 expression and enhances lysosomal activity and antioxidative capacity in adipocytes

**DOI:** 10.1002/2211-5463.13055

**Published:** 2020-12-13

**Authors:** Masaki Kobayashi, Hiromine Yasukawa, Tomoya Arikawa, Yusuke Deguchi, Natsumi Mizushima, Misako Sakurai, Shoichi Onishi, Ryoma Tagawa, Yuka Sudo, Naoyuki Okita, Kyohei Higashi, Yoshikazu Higami

**Affiliations:** ^1^ Laboratory of Molecular Pathology and Metabolic Disease Faculty of Pharmaceutical Sciences Tokyo University of Science Noda Japan; ^2^ Translational Research Center Research Institute of Science and Technology Tokyo University of Science Noda Japan; ^3^ Laboratory of Clinical and Analytical Biochemistry Faculty of Pharmaceutical Sciences Tokyo University of Science Noda Japan; ^4^ Division of Pathological Biochemistry Faculty of Pharmaceutical Sciences Sanyo‐Onoda City University Japan

**Keywords:** adipocyte, lysosome, oxidative stress, SQSTM1, trehalose

## Abstract

Adipocytes, which comprise the majority of white adipose tissue (WAT), are involved in obesity‐related pathology via various mechanisms, including disturbed lysosomal enzymatic activity and accumulation of oxidative stress. Sequestosome 1 (SQSTM1/p62) is an autophagy marker that participates in antioxidative responses via the activation of nuclear factor erythroid‐derived 2‐like 2 (NRF2). Trehalose is a non‐reducing disaccharide reported to suppress adipocyte hypertrophy in obese mice and improve glucose tolerance in humans. We recently revealed that trehalose increases SQSTM1 levels and enhances antioxidative capacity in hepatocytes. Here, to further evaluate the mechanism behind the beneficial effects of trehalose on metabolism, we examined SQSTM1 levels, autophagy, and oxidative stress in trehalose‐treated adipocytes. We initially confirmed that trehalose increases SQSTM1 transcription and protein levels without affecting autophagy in adipocytes. Trehalose also elevated transcription of several lysosomal genes and the activity of cathepsin L, a lysosomal enzyme, independently of the transcription factor EB. In agreement with our data from hepatocytes, trehalose induced the nuclear translocation of NRF2 and the transcription of its downstream antioxidative genes, resulting in reduced cellular reactive oxygen species levels. Moreover, some cellular trehalose was detected in trehalose‐treated adipocytes, implying that extracellular trehalose is taken into cells. These observations reveal the mechanism behind the beneficial effects of trehalose on metabolism and suggest its potential for preventing or treating obesity‐related pathology.

## Graphical abstract

Trehalose is a disaccharide reported to ameliorate adipocyte hypertrophy and improve metabolic state. The present study demonstrated that trehalose augments the levels of SQSTM1, lysosome‐related factors and antioxidative factors, which induces lysosomal activity and antioxidative capacity in adipocytes. These observations reveal a mechanism for the above effects of trehalose.

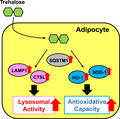

**Abbreviations**
CTSL: cathepsin L
GFP: green fluorescent protein
HO‐1: heme oxygenase‐1
LAMP1: lysosome‐associated membrane protein 1
LC3: microtubule‐associated protein 1 light chain 3
LMNB1: lamin B1
NQO‐1: nicotinamide adenine dinucleotide phosphate quinone dehydrogenase‐1
NRF2: nuclear factor erythroid‐derived 2‐like 2
RFP: red fluorescent protein
ROS: reactive oxygen species
RPS18: ribosomal protein S18
SOD‐1: superoxide dismutase 1
SQSTM1: sequestosome 1
TFEB: transcription factor EB

Obesity is a risk factor for type 2 diabetes and cardiovascular diseases. White adipose tissue (WAT) consists mainly of adipocytes capable of storing energy in the form of triglyceride (TG) and secreting adipokines. Hypertrophic adipocytes are frequently observed in obese individuals and cause chronic inflammation and insulin resistance [1]. The levels of reactive oxygen species (ROS) are elevated in obese WAT, which provokes inflammation and insulin resistance [2]. These findings indicate that the features of adipocytes closely contribute to obesity‐related pathology.

Sequestosome 1 (SQSTM1/p62) protein is a selective autophagy substrate that is generally regarded as a marker of autophagy [3]. Autophagy is a cellular process essential for maintaining cellular homeostasis and occurs via lysosomal clearance [4, 5, 6]. Autophagy is initiated by the formation of autophagosomes, double‐membrane structures containing damaged organelles and proteins. The degradation of the intramembranous contents of autophagosomes occurs via their fusion with acidic lysosomes. The progress of these processes is called autophagic flux. The transcription factor EB (TFEB), a member of the MiT‐TFE family, is a master transcription factor of autophagic and lysosomal genes [7]. We recently demonstrated that high‐fat‐diet‐induced obesity impairs the activity of cathepsin L (CTSL), a major lysosomal enzyme in mouse white adipose tissue, causing inflammatory responses and senescence [8]. SQSTM1 also plays an important role in antioxidative responses through enhancing the transcriptional activity of nuclear factor erythroid‐derived 2‐like 2 (NRF2) [9]. NRF2 is a key transcription factor of antioxidative genes, including *heme oxygenase‐1* (*Ho‐1*), *nicotinamide adenine dinucleotide phosphate quinone dehydrogenase‐1* (*Nqo‐1*), and *superoxide dismutase 1* (*Sod‐1*) [10]. Transcription of these downstream genes is activated by nuclear‐transported NRF2 [11].

Trehalose is a non‐reducing disaccharide composed of two α‐1,1‐linked d‐glucose units, which is reportedly taken into mammalian cells via glucose transporter 8 (GLUT8) [12, 13]. It has been reported that trehalose has preventive effects on obesity‐related pathology. For example, Arai *et al*. [14, 15, 16] demonstrated the suppressive effect of trehalose against high‐fat‐diet‐induced adipocyte hypertrophy and insulin resistance in mice. Additionally, regular trehalose intake does not alter blood glucose levels, but improves glucose tolerance in humans [17]. Previous studies have also shown that trehalose accelerates autophagy and enhances the capacity to remove abnormally aggregated proteins [18, 19]. Very recently, we reported that trehalose upregulates SQSTM1 mRNA and protein levels in mouse hepatocytes in an autophagy‐independent manner [20]. We also showed that trehalose‐induced SQSTM1 upregulation induces NRF2 nuclear translocation, which had antioxidative effects including an increase in the mRNA levels of antioxidative genes and a decrease in ROS [20]. In the present study, to highlight the mechanisms behind the beneficial effects of trehalose on metabolism, we investigated SQSTM1 levels, lysosomal activity, and oxidative stress in trehalose‐treated adipocytes.

## Materials and methods

### Cell culture and differentiation

3T3‐L1 preadipocytes, purchased from RIKEN Bioresource Center (Ibaraki, Japan), were cultured in maintenance medium, Dulbecco's Modified Eagle's Medium (low‐glucose) (Wako, Osaka, Japan) with 10% fetal bovine serum (Thermo Fisher Scientific, Waltham, MA, USA) and 1% penicillin/streptomycin (Sigma‐Aldrich, St. Louis, MO, USA). The differentiation of 3T3‐L1 preadipocytes into mature adipocytes was performed as follows. Preadipocytes were cultured to reach confluence. At confluence, the maintenance medium was changed to adipocyte differentiation medium [the maintenance medium supplemented with 500 μm 3‐isobutyl‐1‐methylxanthine (Sigma‐Aldrich) and 1 μm dexamethasone (Sigma‐Aldrich)], and the cells were then cultured for another 2 days. Subsequently, the adipocyte differentiation medium was changed to adipocyte maturation medium [the maintenance medium supplemented with 10 μg·mL^−1^ insulin (Sigma‐Aldrich) and 50 nm triiodothyronine (Sigma‐Aldrich)], which was exchanged at 1‐day intervals. For this study, 3T3‐L1 adipocytes were differentiated for 7 days and treated with the indicated reagents for 24 h.

### Reagents

Trehalose dihydrate (Wako) was dissolved in culture medium and filtered through 0.22 μm filters (Millipore, Bedford, MA, USA). Chloroquine (Wako) was dissolved in PBS. Rapamycin (LC Laboratories, Woburn, MA, USA) was dissolved in dimethylsulfoxide. 1,1′‐Dimethyl‐4,4′‐bipyridinium dichloride hydrate (paraquat) (Sigma‐Aldrich) was dissolved in PBS. The concentrations of each reagent in the treatment were as follows: 10 μm (chloroquine), 0.5 μm (rapamycin), and 2 mm (paraquat).

### Establishment of 3T3‐L1 cells overexpressing GFP‐LC3 or mRFP‐GFP‐LC3

Plasmids encoding green fluorescent protein (GFP) fused to LC3 (GFP‐LC3) or LC3 tandemly tagged with GFP and red fluorescent protein (RFP) (mRFP‐GFP‐LC3) were obtained from Addgene, Watertown, MA, USA (#21073 or #21074). 3T3‐L1 cells overexpressing GFP‐LC3 or mRFP‐GFP‐LC3 were generated by a retrovirus system using pMXs‐AMNN‐puro‐GFP‐LC3 or pMXs‐AMNN‐puro‐mRFP‐GFP‐LC3 plasmids, as previously reported [20]. Briefly, the vectors were transfected into Plat‐E cells using FuGENE^®^6 (Promega, Madison, WI, USA) following the manufacturer's protocol. Virus‐containing culture supernatants were collected 2 days after transfection and filtered through 0.22 μm filters (Millipore). 3T3‐L1 cells were incubated with virus‐containing medium for 1 day and then selected with 2 μg·mL^−1^ puromycin for 4 days.

### Immunoblotting analysis and antibodies

Cell sampling and immunoblotting were performed as previously described [8]. Signals were detected with Immunostar^®^ LD chemiluminescent substrates (Wako) and a LAS‐3000 image analyzer (Fujifilm, Tokyo, Japan). Signal intensity was quantified using multigauge software (Fujifilm). Antibodies against LC3 (PM036), SQSTM1 (PM045), and Lamin B1 (LMNB1) (PM064) were purchased from MBL (Aichi, Japan). Anti‐TFEB antibody (A303‐673A) was purchased from Bethyl Laboratories Inc. (Montgomery, TX, USA). Anti‐NRF2 antibody (sc‐13032) was purchased from Santa Cruz Biotechnology (Santa Cruz, CA, USA). Anti‐β‐ACTIN (A1978) antibody was purchased from Sigma‐Aldrich. Secondary antibodies included horseradish peroxidase‐conjugated F(ab′)2 fragment of goat anti‐mouse IgG, anti‐rabbit IgG, and anti‐goat IgG (Jackson Immunoresearch, West Grove, PA, USA).

### RNA purification and quantitative real‐time RT‐PCR

Total RNA was extracted from cells using Isogen II (Nippon Gene, Tokyo, Japan), following the manufacturer's protocol. Purified RNA (0.5 μg) was subjected to reverse transcription with Revertra Ace (TOYOBO, Osaka, Japan). Quantitative RT‐PCR was performed using a CFX Connect™ RT‐PCR System (Bio‐Rad, Hercules, CA, USA) with THUNDERBIRD SYBR qPCR Mix (TOYOBO). Quantitative PCR data were processed using a standard curve method. Ribosomal protein S18 (*Rps18*) mRNA expression was used for normalization. The sequences of the primers used in this analysis are shown in Table 1.

**Table 1:** Primers used in the present study.

Gene	Forward (5′ to 3′)	Reverse (5′ to 3′)
*Sqstm1*	GAAGCTGAAACATGGACACTTTG	CATTGGGATCTTCTGGTGGAG
*Lamp1*	TCAGCATCTCCAACCATTCAC	TGAACACACTCTTCCACAGACC
*Ctsl*	TCGGTGACATGACCAATGAG	CACACAACCCTTTTCTCTCCAG
*Ho‐1*	GAACTTTCAGAAGGGTCAGGTG	AGGGAAGTAGAGTGGGGCATAG
*Sod1*	GGATGAAGAGAGGCATGTTGG	TTTGCCCAAGTCATCTTGTTTC
*Nqo‐1*	CGAATCTGACCTCTATGCTATGAAC	GAACTGAAATATCACCAGGTCTGC
*Adiponectin*	TGCCGAAGATGACGTTACTACAAC	CTTCAGCTCCTGTCATTCCAAC
*Mcp1*	CCAGCCAACTCTCACTGAAGC	CTTCTTTGGGACACCTGCTG
*Il‐6*	GCCTTCCCTACTTCACAAGTCC	CAGAATTGCCATTGCACAAC
*Glut8*	CAACTGGTTCATGGCCTTTC	AAATGGGCTGTGACTTGTTCC
*Rps18*	TGCGAGTACTCAACACCAACAT	CTTTCCTCAACACCACATGAGC

### Immunofluorescence analysis

3T3‐L1 cells expressing GFP‐LC3, and those expressing mRFP‐GFP‐LC3, were differentiated on cover slips for 7 days and then treated with the indicated reagents for 24 h. After treatment, the cells were fixed with 4% paraformaldehyde for 10 min, and cover slips were mounted with Dako fluorescence mounting medium (DAKO, Carpinteria, CA, USA). GFP and RFP fluorescence was detected with a TCS SP8 confocal microscope (Leica Microsystems, Wetzlar, Germany).

### Measurement of cellular trehalose levels

3T3‐L1 cells were differentiated on 150‐mm culture dishes and then treated with 50 mm trehalose for 24 h (estimated cell number: 3.3 × 10^4^ per cm^2^). The harvested cells were suspended with 300 μL of methanol : CHCl_3_ : H_2_O (1 : 1 : 1) and centrifuged for 5 min at 13 000 ***g***. After centrifugation, a water layer (approx. 100 μL) was suspended with 200 μL of methanol : CHCl_3_ (1 : 1) and centrifuged for 5 min at 12 000 r.p.m. The resulting water layer was diluted with H_2_O and submitted to analysis using LCMS™‐8050 (Shimadzu, Kyoto, Japan) connected to an LC‐40 chromatograph system. LC separation of trehalose was carried out using an Asahipak NH2P‐40 2D (150 mm × 2.0 mm I.D., 4 μm; Showa Denko K.K., Tokyo, Japan) and the corresponding precolumn (Asahipak NH2P‐50G2A: 10 mm × 2.0 mm I.D., 5 μm; Showa Denko K.K.). Separation of trehalose was performed under isocratic conditions using 0.05% ammonia water/acetonitrile (20/80, v/v) at a constant flow rate of 0.2 mL·min^−1^. Trehalose was analyzed by ESI‐MS in the negative mode. MRM transition was performed at *m/z* 341.00 > 179.05. Optimized values for Q1 Pre Bias, collision energy, and Q3 Pre Bias were 16, 16, and 11 V, respectively. The MS conditions were as follows: nebulizing gas flow of 2 L·min^−1^; heating gas flow of 10 L·min^−1^; interface temperature of 400 °C; DL temperature of 200 °C; heat block temperature of 500 °C; and drying flow of 5 L·min^−1^.

### Isolation of the nuclear fraction

Scraped and washed cell pellets were suspended in buffer A [20 mm HEPES (pH 7.9), 3 mm MgCl_2_, 20 mm KCl, 0.68 m sucrose, 20% glycerol, and 1% Triton X‐100]. After 10 min of incubation on ice, the mixture was centrifuged at 1300 ***g*** for 5 min. The supernatant fraction was further centrifuged at 1300 ***g*** for 10 min. The precipitate was resuspended in buffer A and centrifuged at 1300 ***g*** for 4 min. This step was repeated. The precipitate was obtained for nuclear protein extraction, which was performed using lysis buffer (50 mm Tris/HCl pH 6.8, 2% SDS, and 5% glycerol).

### CTSL activity assay

CTSL activity was assayed as previously reported [8]. In brief, fluorometric analysis with Z‐Phe‐Arg‐MCA (3095‐v; Peptide Institute, Osaka, Japan) was performed using cell pellets resuspended in lysis buffer (352 mm KH_2_PO_4_, 48 mm Na_2_HPO_4_, and 4 mm EDTA, pH 6.0) and incubated on ice for 60 min before centrifugation for 10 min at 2100 ***g***. The supernatant was collected, and protein concentrations were determined using a BCA kit (Pierce, Waltham, MA, USA). The supernatant was then added to the reaction buffer (4 mm DTT in lysis buffer). A total of 100 μL of assay buffer (containing 10 μg of protein) was mixed with 100 μL of substrate buffer (10 μm Z‐Phe‐Arg‐AMC and 10 μm CA074, a selective cathepsin B inhibitor) and incubated at 37 °C for 30 min. Fluorescence was measured using an Infinite F200 Pro Plate Reader (Tecan, Männedorf, Switzerland) with excitation/emission wavelengths of 360/460 nm.

### ROS assay

Differentiated cells (day 7) were pretreated with 50 mm trehalose for 24 h and then treated with 2 mm paraquat for 15 h. Untreated cells were subjected to control treatment with PBS for 24 h after the medium had been changed. Subsequently, the cells were washed with medium and incubated with 10 μm CM‐H2DCFDA (Thermo Fisher) at 37 °C for 30 min. After incubation, the cells were washed twice with medium. The fluorescence intensity was measured using an Infinite F200 Pro Plate Reader (Tecan).

### Statistical analysis

Statistical analysis was performed by Student's *t* test or Tukey's test using BellCurve for Excel software (Social Survey Research Information Co., Ltd., Tokyo, Japan). Data are presented as mean ± standard deviation, and *P* < 0.05 was considered significant.

## Results

**Fig. 1:**
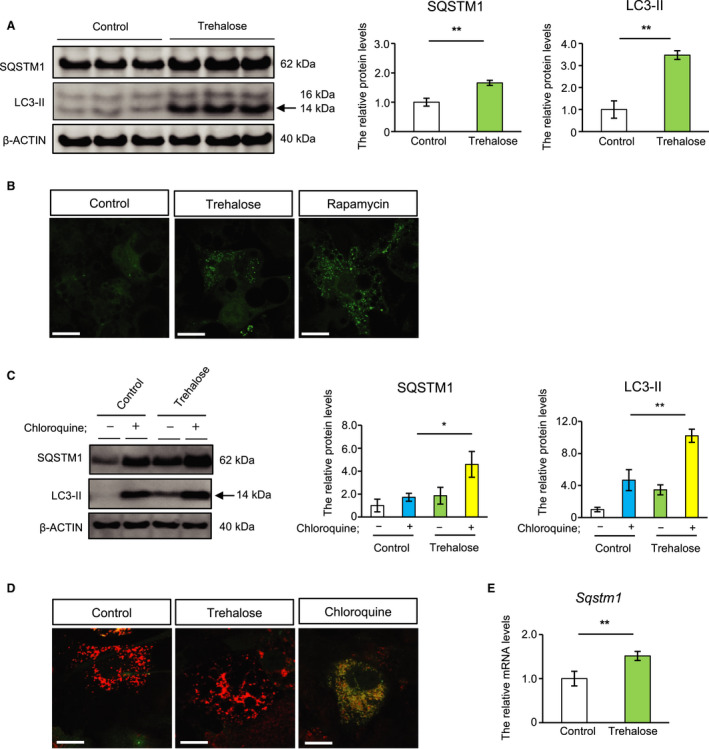
Trehalose increased autophagosome formation and SQSTM1 levels without inhibiting autophagy flux in adipocytes. (A) 3T3‐L1 cells were treated with 50 mm trehalose for 24 h. SQSTM1 and LC3‐II proteins were analyzed by immunoblotting. An arrow shows LC3‐II bands. The right panels show quantitative data for SQSTM1 and LC3‐II (*n* = 3). (B) Fluorescence images of 3T3‐L1 cells expressing GFP‐LC3 treated with 50 mm trehalose or 0.5 μm rapamycin. Green: GFP. (C) 3T3‐L1 cells were treated with 50 mm trehalose, with or without 10 μm chloroquine. SQSTM1 and LC3‐II proteins were analyzed by immunoblotting. The left panel shows a representative image. An arrow shows LC3‐II bands. The right panels show quantitative data for SQSTM1 and LC3‐II (*n* = 3). (D) Fluorescence images of 3T3‐L1 cells expressing mRFP‐GFP‐LC3 treated with trehalose or chloroquine. Red: mRFP; Green: GFP. (E) 3T3‐L1 cells were treated with trehalose. *Sqstm1* mRNA expression levels were analyzed by quantitative RT‐PCR (*n* = 4). Data were normalized to *Rps18* expression. β‐ACTIN was used as a loading control. Control cells were treated with PBS. Values show the mean ± SD. Differences between values were analyzed by Student's *t* test or Tukey's test. Statistical significance is shown as *P < 0.05, **P < 0.01. Scale bars show 20 μm.

We examined SQSTM1 protein levels in differentiated 3T3‐L1 adipocytes treated with trehalose for 24 h. Consistent with our previous results, trehalose treatment significantly increased SQSTM1 protein levels (Fig. 1A and Fig. S1). In parallel with this, the levels of phosphatidylethanolamine‐conjugated microtubule‐associated protein 1 light chain 3 (LC3‐II), used as a marker of autophagosome formation, were also elevated in these cells (Fig. 1A and Fig. S1). Cellular trehalose levels were calculated to be 12.576 nmol per 1 × 10^5^ cells after treatment with 50 mm trehalose for 24 h, while it was not detected in control cells. To evaluate autophagosome formation, we measured GFP dots in 3T3‐L1 adipocytes expressing GFP‐LC3 treated with trehalose. Correlating with LC3‐II abundance, the number of GFP dots in trehalose‐treated cells was comparable to that observed in cells treated with rapamycin, an autophagy inducer (Fig. 1B). In general, simultaneous increases in both SQSTM1 and LC3‐II reflect autophagosome accumulation followed by impaired autophagosome degradation. To determine whether trehalose caused this cellular event, we used chloroquine, a lysosomal inhibitor. The trehalose‐induced increase in SQSTM1 and LC3‐II proteins was observed even in the presence of chloroquine (Fig. 1C), implying that the accumulation of these proteins did not result from the inhibition of autophagosome degradation. To analyze autophagic flux in greater detail, we used 3T3‐L1 adipocytes expressing mRFP‐GFP‐LC3, which allows for the discrimination between early autophagosomes with dual red and green fluorescence, and autolysosomes with only red fluorescence [21]. Trehalose‐treated cells exhibited red puncta equal to those observed in control cells, while chloroquine induced yellow puncta, indicating that trehalose did not impair autophagy flux (Fig. 1D). We have previously shown that trehalose upregulates *Sqstm1* mRNA levels [20]. Hence, we examined *Sqstm1* mRNA levels in 3T3‐L1 adipocytes treated with trehalose. *Sqstm1* mRNA levels were increased by trehalose (Fig. 1E). These results suggest that trehalose can accelerate the formation of autophagosomes without inhibiting autophagic flux in adipocytes.

**Fig. 2:**
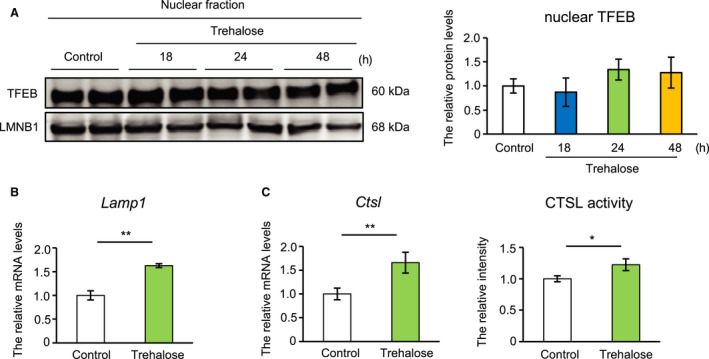
Trehalose upregulated lysosomal gene expression and enzymatic activity in adipocytes (A) Nuclear protein extracts from 3T3‐L1 adipocytes treated with trehalose for 18, 24, and 48 h were prepared and analyzed by immunoblotting. The left panel shows a representative image. The right panel shows quantitative data for TFEB (*n* = 4). LMNB1 was used as a loading control. (B) *Lamp1* mRNA expression levels were analyzed by quantitative RT‐PCR (*n* = 4). (C) *Ctsl* mRNA expression levels were measured by quantitative RT‐PCR (*n* = 4), and CTSL enzymatic activity was analyzed by the selective substrate (*n* = 5). Data of quantitative RT‐PCR were normalized to *Rps18* expression. Control cells were treated with PBS. Values show the mean ± SD. Differences between values were analyzed by Tukey's test or Student's *t* test. Statistical significance is shown as *P < 0.05, **P < 0.01.

We next focused on TFEB, which activates *Sqstm1* transcription [7]. To evaluate TFEB activity, we examined the amount of TFEB protein in the nuclear fraction after treatment with trehalose. The results show that TFEB was not significantly altered by trehalose in the nuclear fraction (Fig. 2A). In contrast, the levels of *lysosome‐associated membrane protein 1* (*Lamp1*), a TFEB target gene, were upregulated by trehalose (Fig. 2B). We previously showed that CTSL contributes to the characteristics of obese adipocytes [8]. Thus, we measured CTSL enzymatic activity and *Ctsl* mRNA levels in trehalose‐treated 3T3‐L1 cells. Similar to *Lamp1* levels, trehalose significantly enhanced CTSL activity and elevated *Ctsl* mRNA levels (Fig. 2C). These results suggest that trehalose may activate lysosomal function in adipocytes in a TFEB‐independent manner.

**Fig. 3:**
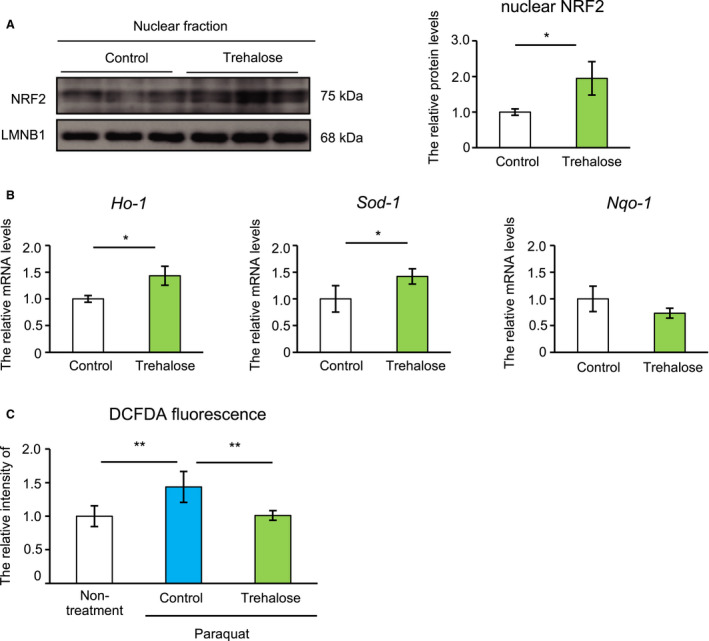
Trehalose suppressed oxidative stress in adipocytes. (A) Nuclear protein extracts from trehalose‐treated cells were prepared and analyzed by immunoblotting. The right panel shows quantitative data for NRF2. LMNB1 was used as a loading control. (B) *Ho‐1*, *Sod‐1*, and *Nqo‐1* mRNA expression levels were analyzed by quantitative RT‐PCR (*n* = 4). Data were normalized to *Rps18* expression. (C) Intracellular ROS levels in 3T3‐L1 adipocytes (treated as indicated) were analyzed with CM‐H2DCFDA (*n* = 4). Control cells were treated with PBS. Values show the mean ± SD. Differences between values were analyzed by Student's *t* test or Tukey's test. Statistical significance is shown as *P < 0.05, **P < 0.01.

We examined the amount of NRF2 protein in the nuclear fraction and the expression levels of NRF2 downstream gene targets, including *Ho‐1*, *Nqo‐1*, and *Sod‐1*. As expected, trehalose significantly increased NRF2 protein in the nuclear fraction and *Ho‐1* and *Sod‐1* expression (Fig. 3A,B), but *Nqo‐1* levels were unchanged (Fig. 3B). To investigate the antioxidative capacity of trehalose, we measured cellular ROS levels induced by paraquat, a general ROS generator, using the DCFDA fluorescent probe as an indicator of ROS [22]. Pretreatment with trehalose significantly prevented paraquat‐induced DCFDA fluorescence (Fig. 3C). These results suggest that trehalose suppresses oxidative stress.

## Discussion

We demonstrate here that trehalose increases the abundance of SQSTM1 proteins and induces autophagosome formation in adipocytes. Additionally, trehalose‐induced augmentation of SQSTM1 occurs at the mRNA level. This is consistent with previous reports showing that trehalose upregulates *Sqstm1* levels in mouse hepatoma cells or motor neuron cells [20, 23]. In the latter report, trehalose was also demonstrated to enhance the nuclear translocation of TFEB in a time‐dependent manner, which was identified as the mechanism of trehalose‐induced *Sqstm1* upregulation [23]. However, our results showed that trehalose does not alter TFEB abundance in the nuclear fraction of 3T3‐L1 cells, even though *Lamp1* and *Ctsl* mRNA levels are upregulated. In other words, TFEB activity did not correlate with the transcription of *Sqstm1* and lysosomal genes in trehalose‐treated adipocytes. In addition to TFEB, the MiT‐TFE family includes MITF, TFEC, and TFE3 [24]. In particular, TFE3 regulates the transcription of lysosome‐ and autophagy‐related genes, which overlap with downstream TFEB targets [25]. Therefore, we hypothesize that TFE3 could be involved in trehalose‐induced lysosomal activation in adipocytes.

Previous reports, including ours, have demonstrated the involvement of trehalose in oxidative stress. For example, Tang *et al*. [26] provided evidence that trehalose prevents oxidative stress‐induced mitochondrial injury in chondrocytes. We previously showed that trehalose induces the nuclear translocation of NRF2 via increased SQSTM1 expression, thereby reducing paraquat‐induced ROS levels in hepatocytes [20]. We also demonstrated that trehalose‐induced NRF2 activation is mediated by SQSTM1 [20]. Likewise, trehalose‐treated adipocytes exhibited enhanced nuclear NRF2 translocation and increased expression of NRF2 downstream target genes, *Ho‐1* and *Sod‐1*, leading to ROS suppression in adipocytes. Given our reported results, it is suggested that SQSTM1 upregulation may be involved in these trehalose‐mediated effects. However, our results presented here showed that trehalose did not upregulate *Nqo‐1* mRNA levels as it did in hepatocytes [20]. Why these transcriptional responses differ between cell types is unclear, but may be related to simultaneous negative regulation. Dhakshinamoorthy and Jaiswal have demonstrated that MafG, MafK, and c‐Maf negatively regulate *Nqo‐1* promoter activity even under conditions of NRF2 overexpression [27, 28]. Further analysis will be required to clarify the association between these factors and trehalose‐mediated effects.

In our analysis, cellular trehalose levels were calculated to be about 12 nmol/10^5^ cells in 3T3‐L1 adipocytes, implying that a certain amount of exogenous trehalose was taken up into them. Given the differences in experimental conditions, including the type of cell, treatment time, and concentration, this value is roughly consistent with a previous report [29]. We also confirmed the expression of the *Glut8* gene, encoding a trehalose transporter, in both 3T3‐L1 preadipocytes and adipocytes (Fig. S2). These findings suggest that transported intracellular trehalose itself might exert the effects observed in trehalose‐treated adipocytes.

Our data show that trehalose increases SQSTM1 abundance, without inhibiting autophagy. Trehalose also enhances lysosomal activity and antioxidative capacity in adipocytes. These results are expected to represent a mechanism behind the beneficial effects of trehalose on metabolism. However, trehalose failed to affect the levels of adipokines, such as *Adiponectin*, an anti‐inflammatory adipokine, *Mcp1* (*Ccl2*; chemokine (C‐C motif) ligand 2) and *Il‐6* (interleukin‐6), pro‐inflammatory adipokines, which are generally implicated in oxidative stress in adipocytes (Fig. S3). A possible explanation for this result is that the antioxidative effects of acute treatment with trehalose may be insufficient to induce the changes in adipokines. Evaluation of the chronic effects of trehalose will require experiments using animal models. In conclusion, the present study reveals a mechanism behind the beneficial effects of trehalose and helps to demonstrate that trehalose could be a natural compound to prevent or treat obesity‐related pathology.

## Conflict of interest

The authors declare no conflict of interest.

## Author contributions

MK and YH conceived the study, designed the experiments, and wrote the manuscript. HY predominantly performed the experiments and wrote the manuscript under the leadership of MK. SO and KH contributed to the measurement of cellular trehalose levels. RT, NO, and YS contributed to the discussion. TA, YD, NM, and MS assisted the experiments.

## Supporting information


**Fig. S1.** 3T3‐L1 cells were treated with the indicated concentrations of trehalose for 24 h. SQSTM1 and LC3‐II proteins were analyzed by immunoblotting. An arrow shows LC3‐II bands. β‐ACTIN was used as a loading control.
**Fig. S2.** 3T3‐L1 cells were treated with 50 mM trehalose for 24 h. *Adiponectin*, *Mcp1* and *Il‐6* mRNA levels were analyzed by quantitative RT‐PCR. *Rps18* was used as a housekeeping gene. Values show the mean ± SD.
**Fig. S3.** *Glut8* expression was detected in both 3T3‐L1 preadipocytes (pre) and mature adipocytes (mature) by semi‐quantitative RT‐PCR. Semi‐quantitative RT‐PCR was performed using Blend‐taq plus (Toyobo, Osaka, Japan) and the primers for shown genes. *Rps18* was used as a housekeeping gene.

## Data Availability

The data will be available from the corresponding author upon reasonable request.
