# Supplementary material for: Trehalose induces SQSTM1/p62 expression and enhances lysosomal activity and antioxidative capacity in adipocytes
Source: FEBS Open Bio. 2020 Dec 13;11(1):185–94. doi: 10.1002/2211-5463.13055 (PMC7780112; doi:10.1002/2211-5463.13055)
Supplement: Supplementary file 1 — Fig. S1. 3T3‐L1 cells were treated with the indicated concentrations of trehalose for 24 h. SQSTM1 and LC3‐II proteins were analyzed by immunoblotting. An arrow shows LC3‐II bands. β‐ACTIN was used as a loading control. Fig. S2. 3T3‐L1 cells were treated with 50 mM trehalose for 24 h. Adiponectin, Mcp1 and Il‐6 mRNA levels were analyzed by quantitative RT‐PCR. Rps18 was used as a housekeeping gene. Values show the mean ± SD. Fig. S3. Glut8 expression was detected in both 3T3‐L1 preadipocytes (pre) and mature adipocytes (mature) by semi‐quantitative RT‐PCR. Semi‐quantitative RT‐PCR was performed using Blend‐taq plus (Toyobo, Osaka, Japan) and the primers for shown genes. Rps18 was used as a housekeeping gene. [file FEB4-11-185-s001.pdf]

Supplementary Figure 1

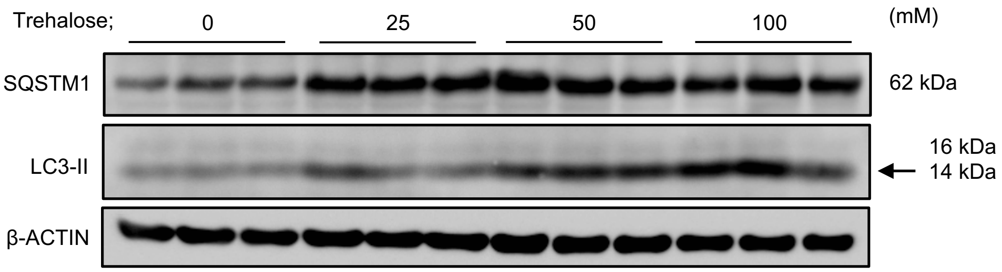

Fig. S1. 3T3-L1 cells were treated with the indicated concentrations of trehalose for 24 h. SQSTM1 and LC3-II proteins were analyzed by immunoblotting. An arrow shows LC3-II bands. β-ACTIN was used as a loading control.

Supplementary Figure 2

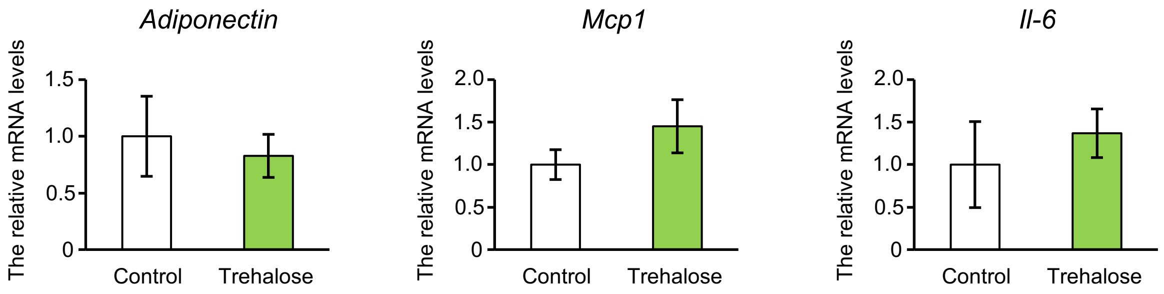

Fig. S2. 3T3-L1 cells were treated with 50 mM trehalose for 24 h. *Adiponectin*, *Mcp1* and *Il-6* mRNA levels were analyzed by quantitative RT-PCR. *Rps18* was used as a housekeeping gene. Values show the mean ± SD.

Supplementary Figure 3

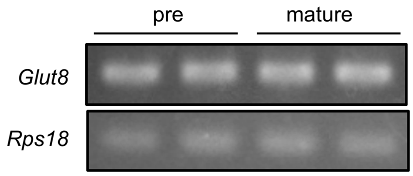

Fig. S3. *Glut8* expression was detected in both 3T3-L1 preadipocytes (pre) and mature adipocytes (mature) by semi-quantitative RT-PCR. Semi-quantitative RT-PCR was performed using Blend-taq plus (Toyobo, Osaka, Japan) and the primers for shown genes. *Rps18* was used as a housekeeping gene.
